# Describing diagnosis (MAT + PCR) and control (vaccine + streptomycin sulfate) of a leptospirosis outbreak in dairy cows with reproductive disorders

**DOI:** 10.1590/1984-3143-AR2025-0011

**Published:** 2025-10-17

**Authors:** Matheus Aguiar Stein, Nayara Bastos Costa, Glaucenyra Cecília Pinheiro da Silva

**Affiliations:** 1 Programa de Pós-graduação Stricto Sensu em Biociência Animal, Universidade de Cuiabá, Cuiabá, MT, Brasil; 2 Programa de Pós-graduação em Biociência Animal, Universidade Federal de Jataí – UFJ, Jataí, GO, Brasil

**Keywords:** bovines, control, diagnosis, reproduction, *Leptospira* spp

## Abstract

Leptospirosis is a zoonosis caused by bacteria of the genus *Leptospira*. In cattle, the infection mainly manifests in the genital form. However, there are still few studies about this manifestation. The aim of this study was to describe the control of an outbreak of leptospirosis in dairy cows with reproductive disorders, through the combination of diagnostic methods and the integration of vaccination with antibiotic therapy. Between December 2022 and April 2023, 17 cows presented reproductive disorders. After the outbreak, two consecutive blood collections and one cervicovaginal mucus (CVM) collection were carried out. The blood samples were tested by the microscopic seroagglutination test (MAT), using a collection of antigens with eight strains of *Leptospira* and a cutoff point ≥ 1:200. The CVM samples were analyzed by polymerase chain reaction (PCR), with the *lip*L32 gene as target. The control was carried out with the CattleMaster® GOLD FP 5/L5 vaccine, in addition to the application of streptomycin (25 mg/kg) in positive cows. After one year of sanitary management, the CVM PCR was repeated to evaluate the effectiveness of the integrated control. In serology, 58.8% (10/17) of the cows were reactive, with 100% (10/10) for the serogroup Sejroe. In the molecular analysis, 58.8% (10/17) of the cows were positive. When combining the two methods, the result was 82.3% (14/17) reagent/positive. After the integrated control, 0.0% (0/17) of cows were positive. It was concluded that the outbreak was related to bovine leptospirosis. Furthermore, the combination of diagnostic methods and integrated control proved to be efficient.

## Introduction

Leptospirosis is a zoonosis of global importance, occurring most frequently in tropical regions, such as Brazil (Bradley and Lockaby, 2023). The infection is caused by pathogenic bacteria belonging to the genus *Leptospira*. Among domestic animals, bovines stand out as the most affected species, being characterized as maintenance hosts for strains of serogroup Sejroe. The host-parasit relationship between these strains and cattle makes the infection to be subclinical and chronic (Ellis, 2015).

According to researchers, the main strains that infect cattle are *Leptospira borgpetersenii* serovar Hardjo (Hardjobovis) and *Leptospira interrogans* serovar Hardjo (Hardjoprajitno), while in Brazil strains of *Leptospira santarosai* serovar Guaricura are important too (Ellis, 2015; Aymée et al., 2022a, b). The serogroup Sejroe has a preference for colonizing the uterus, leading to reproductive disorders, such as, birth of weak calves, estrus repetition, and pregnancy losses (Aymée et al., 2024a; Pinto et al., 2020). It has been demonstrated that long term colonization by leptospires occurs independently of renal infection, leading to a chronic silent disease that specifically affects the genital tract, named Bovine Genital Leptospirosis (BGL) (Loureiro and Lilenbaum, 2020).

Bovine leptospirosis is widely disseminated in Brazil, with 35.3% of cattle being reactive for *Leptospira* spp. (Furquim et al., 2021). The state of Rio de Janeiro has a seroprevalence of 38.3% for bovine leptospirosis and Sejroe as the predominant serogroup (Martins and Lilenbaum, 2013). Among the parameters used to evaluate dairy cattle farming, reproductive efficiency is a direct indicator of the health and well-being of the herds. In Brazil, different cases of leptospirosis in dairy cattle have already been reported, and there were cases that infertility reduced reproductive efficiency, leading to great economic losses of up to 84% of the annual gross margin (Carvalho et al., 2024).

Bovine leptospirosis can be diagnosed by different tests, the microscopic agglutination test (MAT) and polymerase chain reaction (PCR) are the main tests used in the field. MAT is a serological method that detects antibodies against leptospires, and it is defined as gold standard for serological diagnosis of leptospirosis (WOAH, 2021). Although useful at the herd level and in the diagnosis of the acute clinical form of leptospirosis, MAT is not recommended for detection of silent carriers of leptospires (Aymée et al., 2024b). The molecular methods are the most important tools for diagnosis of the chronic silent leptospirosis on domestic animals. The detection of the *lip*L32 gene by conventional polymerase chain reaction (PCR) has been widely applied for primary detection of leptospiral DNA on urinary and/or reproductive tract of domestic animals (Di Azevedo and Lilenbaum, 2021). The usage of cervicovaginal mucus (CVM) samples in PCR was demonstrated to be more suitable than the usage of urine to detect leptospiral DNA, so CVM is characterized as a representation of the uterine environment (Aymée et al., 2025). The implementation of a two-step protocol based on serological screening of the herds by MAT and an individual confirmatory diagnosis of the animals by PCR of genital samples, preferentially CVM, allows the implementation of an efficient control program against leptospirosis outbreaks in dairy herds (Loureiro and Lilenbaum, 2020).

One of the strategies acquired by leptospires is hiding in tissues and organs, such as genital tract, evading host imune system (Chin et al., 2020). Vaccination is the most used and lowest-cost measure to control bovine leptospirosis, but in cases of chronic infections, as leptospires are no longer present in the bloodstream, it does not prevent colonization of genital tract, so infected animals can continue to eliminate leptospires (Martins et al., 2022). On the other hand, antibiotic therapy reduces the elimination of leptospires in body fluids, preventing animals from the same herd from becoming infected (Bautista et al., 2022). As suggested by Martins and Lilenbaum (2017), the integration of vaccination and antibiotic therapy in the control of bovine leptospirosis could allow each on to overcome the weaknesses of the other.

Thus, the objective of the present study was to describe the control of an outbreak of leptospirosis in dairy cows with reproductive disorders, in the municipality of Valença, Rio de Janeiro, Brazil, through the combination of diagnostic methods and the integration of vaccination with antibiotic therapy.

## Methods

The study was carried out according to the guidelines of the ethical committee of the University of Cuiabá. All experimental protocols were approved by the Ethics Committee in the Use of Animals (CEUA/UNIC) of the University of Cuiabá, registered under nº 004/2024.

### Study area

The outbreak occurred between December 2022 and April 2023, on a dairy farm in the municipality of Valença (22° 14' 46” South, 43° 42' 11” West), located in the south of the state of Rio de Janeiro, Brazil. The municipality has an area of 1,300.767 km^2^, being at an altitude of 551 m (Cidade-Brasil, 2021). The climate of this region is characterized as tropical, with average temperatures throughout the year of 21.6 °C, with maximum temperatures of 28.2 °C and minimum temperatures of 15.6 °C. The accumulated precipitation throughout the year is 1,231.4 mm, with the greatest accumulation of precipitation between the months of November and March (INMET, 2010).

Valença has the second largest cattle herd in the state of Rio de Janeiro, totaling 106,317 heads, which 18,145 are dairy cows. Milk production in the municipality totals 27,417,000 L, also being the second largest in the state, with income of R$ 75,396,000.00 (IBGE, 2022).

### Herd and animals

The farmer reported that of 36 females of reproductive age (≥ two years old), six (16.7%) presented estrus repetition, four (11.1%) abortions, four (11.1%) birth of weak calves and three (8.3%) stillbirths, totaling 17 cows with reproductive disorders. The herd had never been vaccinated against leptospirosis. Furthermore, no clinical signs other than the reproductive disorders were observed. The management system adopted by the study dairy farm was extensive, so the herd fed on pasture, drank water from waterholes and the reproduction was based on natural breeding.

### Sample collection

Blood samples were taken only from cows with reproductive disorders in the farm (*n* = 17) in April 2023, approximately one month after the last disorders were noted. Two months after the first visit, a new blood collection was performed, and cervicovaginal mucus samples were also collected. Blood samples were colected by venipuncture of coccygeal vein into 10-mL vaccum tubes without anticoagulant. CVM samples were collected with sterile swabs directly from vaginal fornix and then stored in sterile 15-mL Falcon tubes (Loureiro and Lilenbaum, 2020). Approximately one year after the start of sanitary management, a second collection of CVM samples from the 17 cows were performed, with the aim of verifying the efficiency of the integrated control in reducing animals with genital leptospirosis. All collected samples were immediately refrigerated between 2 °C and 8 °C and transported on the same day to the Universidade Federal Fluminense in Rio de Janeiro. The samples were processed and submitted for serology and molecular analysis on the next day after each collection.

### Serology

The serological diagnosis was performed using the microscopic agglutination test that is characterized as a gold standard method for serodiagnosis of leptospirosis (WOAH, 2021). It was used an antigen panel composed by eight reference strains: *Leptospira borgpetersenii* serovar Autumnalis, *Leptospira borgpetersenii* serovar Hardjo (Hardjobovis), *Leptospira interrogans* serovar Icterohaemorrhagiae (st. RGA), *Leptospira interrogans* serovar Pomona, *Leptospira interrogans* serovar Copenhageni, *Leptospira interrogans* serovar Hardjo (Hardjoprajitno), *Leptopsira interrogans* serovar Canicola and *Leptospira santarosai* serovar Guaricura. The strains selected for the antigen panel are known to be prevalent in Brazil (Pinto et al., 2016). The serum titer was considered as the reciprocal of the greatest dilution that presented at least 50% agglutinated leptospires. The individuals were considered reactive when the titer was 200 or more, as suggested for tropical endemic areas (Pinto et al., 2016).

### DNA extraction and molecular detection

DNA was extracted from CVM samples using a DNeasy® Blood & Tissue Kit (Qiagen, California, USA) according to the manufacturer’s instructions. PCR was performed targeting the *lip*L32 gene common in pathogenic leptospires (Stoddard et al., 2009), using primers and conditions described in Hamond et al. (2014). Platinum® Taq DNA Polymerase (Invitrogen, Massachusetts, USA) was used for the amplification reactions. For each set of samples, ultrapure water was used as negative control in all reactions, while 10 fg of DNA extracted from *L. interrogans* serovar Copenhageni (Fiocruz L1-130) was used as the positive control. PCR products were analyzed by electrophoresis in 1.5-2% agarose gel and visualized under ultravioleta light after gel red staining.

### Control

Together with the second sample collection, the 17 cows were vaccinated with the commercial inactivated vaccine CattleMaster® GOLD FP 5/L5 (Zoetis, New Jersey, USA), 5 mL subcutaneously, second booster dose after 21 days and anual revaccination with a single dose. Seroreactive or positive cows for CVM PCR were treated 10 days after the second visit with the antibiotic Estreptomax® (Ourofino, São Paulo, BR), 25 mg of streptomycin sulfate per kg of body weight, intramuscularly and a single dose.

### Statistical analysis

Data relating to the identification of cows (1 to 17), respective reproductive disorders (estrus repetition, abortion, stillbirth and birth of weak calf), and serology and molecular analysis results were all cleaned, arranged and tabulated by Microsoft Excel 2016. Thereafter, the statiscal analysis were performed using Epi Info 7.2 software (CDC, USA). The results were expressed as percentage, allowing descriptive analysis of the predominant reproductive disorder, the level of detection of reactive/positive cows and the efficiency of bovine genital leptospirosis control.

## Results

Among the 17 cows with reproductive disorders, six (35.3%) presented estrus repetition, four (23.5%) abortions, four (23.5%) birth of weak calves and three (17.6%) stillbirths.

Of the 17 cows, eight (47.0%) were reactive at first MAT, whereas three (17.6%) were reactive in the second MAT, with titers of 200. The predominant serogroup was Sejroe, with 100.0% of reactive cows showing serological reactions against it. When comparing the results of the two consecutive MAT, two animals (cows 4 and 5) were reactive in the second, but not reactive in the first. Therefore, when adding the results of the first and second MAT, it was found that 10 cows (58.8%) were reactive.

In the first PCR, of the 17 cows, ten (58.8%) were positive and of these, four (cows 6, 8, 10 and 12) were not reactive in the first and second MAT. Thus, adding the results of consecutive MAT with those of PCR, it was determined that fourteen cows (82.3%) were reactive/positive. The results of combined diagnostic methods (82.3%) independently compared to the results of serological method (58.8%) and those of the molecular method (58.8%), presented an increase of 40.0% in the number of reactive/positive animals.

Approximately, one year after the start of the integrated control, a second CVM PCR was performed. From 10 postive cows (58.8%) in the first PCR, it dropped to zero positive cows (0.0%) in the second one, as described in Table 1, a reduction of 100.0%.

**Table 1:** Results of diagnostic tests in 17 cows with reproductive disorders in a leptospirosis outbreak in the municipality of Valença, Rio de Janeiro, Brazil.

**Cows**	**Reproductive disorders**	**1st serology/serogroup/titer**	**2nd serology** * **/serogroup/titer**	**1st** **molecular analysis**	**2nd** molecular analysis **
1	Birth of Weak Calves	+/Sejroe/200	-	+	-
2	Abortion	+/Sejroe/200	-	-	-
3	Abortion	+/Sejroe/200	-	+	-
4	Estrus repetition	-	+/Sejroe/200	+	-
5	Estrus repetition	-	+/Sejroe/200	-	-
6	Abortion	-	-	+	-
7	Birth of Weak Calves	+/Sejroe/200	-	+	-
8	Abortion	-	-	+	-
9	Estrus repetition	-	-	-	-
10	Stillbirth	-	-	+	-
11	Estrus repetition	+/Sejroe/200	-	-	-
12	Stillbirth	-	-	+	-
13	Estrus repetition	-	-	-	-
14	Stillbirth	+/Sejroe/200	-	+	-
15	Estrus repetition	+/Sejroe/200	-	+	-
16	Estrus repetition	+/Sejroe/200	+/Sejroe/200	-	-
17	Estrus repetition	-	-	-	-

* 60 days after the first serology; **1 year after the start of integrated control.

**Figure 1:**
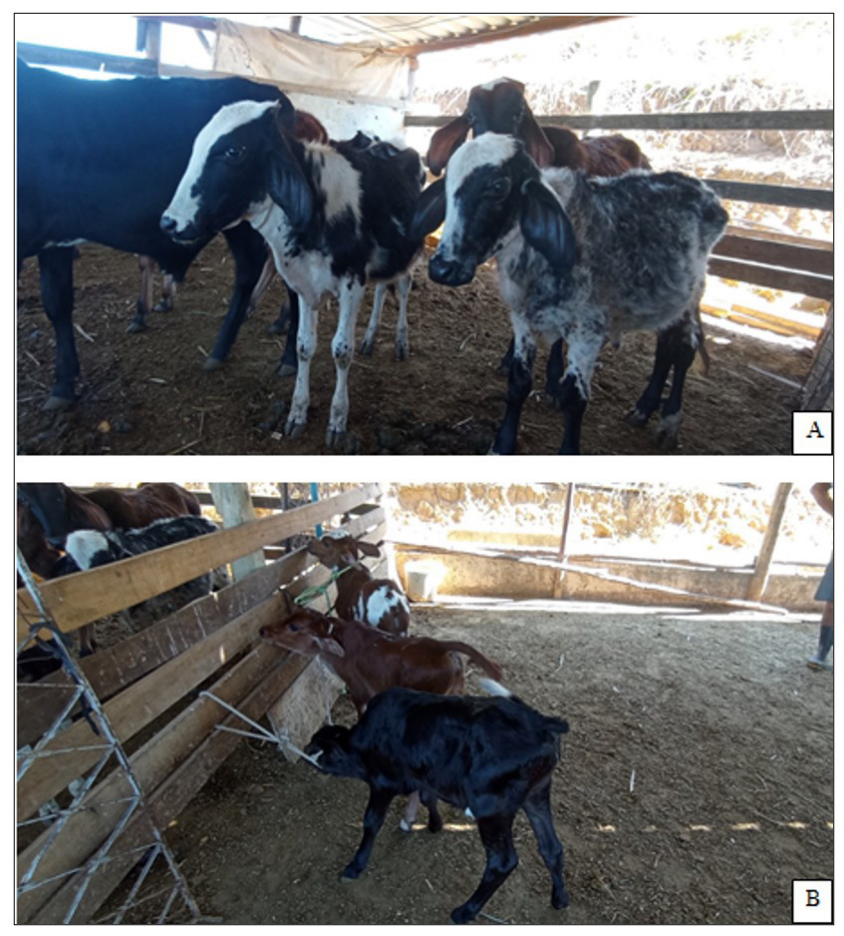
Health of calves born before and after the implementation of integrated control of a leptospirosis outbreak in the municipality of Valença, Rio de Janeiro, Brazil. (A) Calves born before the integrated control; (B) Calves born after the integrated control.

In addition, the owner of the study dairy farm reported that one year after the start of the integrated control, only three cows remained with reproductive disorders. One of these had an abortion and the other two had estrus repetition. The other 14 cows apparently presented a clinical cure of reproductive disorders, with 11 cows got pregnant and three cows gave birth to healthy calves, as is shown in Figure 1.

## Discussion

The clinical aspects of bovine leptospirosis indicate that it is an infectious disease involving the reproductive system (Ellis 2015). The adapted strains, mainly those belonging to the serogroup Sejroe are responsible for colonizing the reproductive tract of bovine females, leading to a specific syndrome named Bovine Genital Leptospirosis, which main sings are pregnancy losses, ranging from embryonic death (more frequently) to late-term abortions (Aymée et al., 2024a). In this study, 47.2% (17/36) of the cows presented estrus repetition, abortions, birth of weak calves or stillbirths. Moreover, estrus repetition was the predominant reproductive disorder with 35.3% (6/17) of cases. These findings are in line with the results demonstrated by Libonati et al. (2018), which identified estrus repetition in dairy herds from Rio de Janeiro, Brazil, as the most commonly reported finding associated with reproductive disorders, which was also strongly associated with infection by leptospires, especially those belonging to serogroup Sejroe.

Of the eight strains of *Leptospira* used in serology, only three responded with titers = 1:200, *Leptospira interrogans* serovar Hardjo (Hardjobovis), *Leptospira borgpeterseni* serovar Hardjo (Hardjoprajitno) and *Leptospira santarosai* serovar Guaricura. These three strains are known to cause a chronic and silent infection in cattle, which embryonic death and consequent estrus repetition are the main signs (Oliveira et al., 2022). This fact may be related to the large number of cows presenting reproductive disorders in the present study, especially the predominance of estrus repetition. Although the region’s climate is characterized by high rainfall and high temperatures, namely the ideal conditions for survival and spread of leptospires, the infection was probably not related to environmental transmissition, because only strains not adapted to cattle are influenced by these factors. In this case, as Hardjobovis, Hardjoprajitno, and Guaricura are strains adapted to cattle, bovine-to-bovine transmission was probably responsible for the outbreak (Correia et al., 2017). The serogroup Sejroe predominated in 100.0% (10/10) of MAT reactive cows. This result was already expected, because serogroup Sejroe is known to preferentially infect cattle, and it is the predominant serogroup in cattle in the state of Rio de Janeiro, Brazil (Martins and Lilenbaum, 2013; Sohm et al., 2023).

In the two consecutive MAT, 10 (58.8%) cows were reactive, and a discrepancy was observed between the results of the two tests, with eight animals reactive (47.0%) in the first MAT and three animals reactive (17.6%) in the second one. The reduction in the number of reactive animals between the two serologies may be explained by the chronic aspect of the serogroup Sejroe infection on cattle, which after one week from the beginning of the infection, the leptospires migrate from bloodstream to preference organs, such as, genital tract (Aymée and Lilenbaum, 2024; Aymée et al., 2024a). Then, at the end of the leptospiremia and with the migration of the leptospires to colonization sites, there is a reduction in antibody production, so the titers that were previously high may become low (Ellis 2015). As opposed to the general reduction in the titers, two animals (cow 4 and 5) were not reactive in the first MAT, but became reactive in the second one. They may have been infected in the interval between the two consecutive tests, because the study farm presents risk factors, such as, waterholes and natural breeding, facilitating bovine-to-bovine transmission.

In the first PCR, 10 (58.8%) cows were positive. It comproves the chronic characteristic of bovine genital leptospiroses, since the first collection of CVM was performed approximately two months after the end of the outbreak, in other words, the leptospires had already migrated to preference organs, in this case the genital tract (Ellis, 2015). The detection of leptospiral DNA in CVM by PCR together with the fact that the predominant serogroup in the two consecutive MAT was Sejroe reinforces that this serogroup has a preference for the genital tract of bovines (Borges et al., 2024). Regarding the cows 6, 8, 10 and 12 that were positive in the PCR, but were not reactive in the two consecutive serologies, it indicates that MAT is a limited technique to diagnose bovine genital carriers individualy, and if only MAT is applied, genital carriers may pass undetected (Aymée et al., 2024b). The fact that cows with reproductive disorders, when they are in the chronic phase of leptospirosis, may present negative results in serology and positive in molecular analysis was described by Aymée et al. (2021), who evaluated the ocurrence of leptospirosis in cattle with low reproductive efficiency. It was demonstraded that from nine cows with low reproductive efficiecy, three (33.3%) were reactive in the MAT and six (66.7%) had positive genital samples in the PCR. Furthermore, three cows that were non reactive in the MAT, they were positive in the PCR.

When evaluating the diagnostic methods individually, it appears that the MAT resulted in 58.8% (10/17) of reactive cows, the same result as PCR. Nevertheless, evaluating the results of the two methods combined, the percentage of reactive/positive cows was 82.3% (14/17). The increase in the number of individuals diagnosed with leptospirosis is related to the fact that four cows reactive in MAT were not positive in PCR (cows 2, 5, 11 and 16), as four cows that were positive in PCR were not reactive in MAT (cows 6, 8, 10 and 12). It’s may be related to the different phases of the infection detected by each of the diagnose methods, since MAT detected reactive individuals in leptospiremic phase, as PCR detected positive individuals in the chronic phase of the infection (Aymée and Lilenbaum, 2024).

The integration of vaccination with antibiotic therapy was efficient in controlling the outbreak studied, because from 58.8% (10/17) of positive cows in the first PCR, after one year of the begnning of integrated control, this result reduced to 0.0% (10/17) of positive cows in the second PCR. Furthermore, according to the herd owner, there was a significant reduction in reproductive disorders, indicating the clinical cure of leptospirosis. Only three cows remained with disorders, and they were probably reinfected by leptospiras from contaminated water sources, since the herd drinks water from waterholes, where infected cows may urinate. Despite not presenting a diagnosis by PCR of genital samples, Martins et al. (2010) conducted a study with a dairy herd that had poor reproductive performance related to leptospirosis, in the state of Rio de Janeiro, Brazil. This study showed that the implementation of a control program based on treatment with 25 mg/kg streptomycin, and a comercial vaccine containing Hardjo bacterin considerably reduced the number of seropositive animals for leptospirosis and improved the reproductive performance, although a small percentage (12%) of the heard remained with reproductive failure. These results are in line with the presente study, presenting the integrated control as a satisfactory strategy to reduce reproductive disorders caused by bovine leptospirosis, however was not sufficient to eliminate the ocurrence of reinfections from the environment.

Although vaccination is the most recommended control measure in preventing and reducing the clinical signs of leptospirosis, it can not combat like antibiotic therapy the colonization of kidneys and genital tract. Therefore, the elimination of leptospires is controlled only by antibiotic therapy. These results confirm the statements of Martins and Lilenbaum (2017), that the integration of vaccination and antibiotic therapy in the control of bovine leptospirosis allows each of the two control strategies to overcome the weaknesses of the other, and the environmental management should be carefully considered in tropics. Biosafety measures, such as hygiene programs involving the access by cattle to clean water and soil sealing, reducing co-grazing with other domestic species particularly with pigs, as well as quarantine and isolation of infected animals, shoud be apllied in cases of tropical regions and/or endemic locations for bovine leptospirosis, like the one in the present study (Dobson, 1974; Waldmann, 1990; Lilenbaum and Souza, 2003; Schoonman and Swai, 2010; Mughini-Gras et al., 2014). In the literature, there are no studies that prove by statistical analysis the reduction of positive cows in PCR after the integrated control. However, there are three studies that demonstrate the clinical cure of bovine leptospirosis after the integration of vaccination and antibiotic therapy to control leptospirosis outbreaks in dairy herds (Martins et al., 2010; Mughini-Gras et al., 2014; Pimenta et al., 2019).

## Conclusion

Thus, the outbreak studied was related to bovine leptospirosis, caused by leptospires from serogroup Sejroe. Among the reproductive disorders reported, estrus repetition was the most predominant. The combination of MAT and PCR compared to the application of each of the two diagnostic methods individually, allowed the detection of a higher number of reactive/positive individuals. The integration of vaccination with antibiotic therapy proved to be efficient in controlling cows positive for leptospires infection in the genital tract.

## Data Availability

Research data is available in a repository.

## References

[B001] Aymée L, Gregg WRR, Loureiro AP, Di Azevedo MIN, Pedrosa JS, Melo JSL, Carvalho-Costa FA, Souza GN, Lilenbaum W (2021). Bovine Genital Leptospirosis and reproductive disorders of live subfertile cows under field conditions. Vet Microbiol.

[B002] Aymée L, Di Azevedo MIN, Borges ALDSB, Carvalho-Costa FA, Lilenbaum W (2022). *Leptospira* spp. strains associated with Bovine Genital Leptospirosis (BGL). Microb Pathog.

[B003] Aymée L, Di Azevedo MIN, Pedrosa JS, Melo JSL, Carvalho-Costa FA, Lilenbaum W (2022). The role of *Leptospira santarosai* serovar guaricura as agent of bovine genital leptospirosis. Vet Microbiol.

[B004] Aymée L, Lilenbaum W (2024). Comments on the sensitivity variation of serology to diagnose bovine leptospirosis: facing the chronic infection. Prev Vet Med.

[B005] Aymée L, Mendes J, Lilenbaum W (2024). Bovine genital leptospirosis: an update of this important reproductive disease. Animals.

[B006] Aymée L, Borges ALDSB, Souza GN, Lilenbaum W (2024). Is microscopic agglutination test a reliable method for diagnosing the bovine genital leptospirosis syndrome?. Vet Res Commun.

[B007] Aymée L, Reis L, Soares AC, Souza GN, Lilenbaum W (2025). Detecting *Leptospira* spp. infection in cows by PCR: what is the best sample to test?. Theriogenology.

[B008] Bautista JM, Aranda Estrada M, Gutiérrez Olvera L, Lopez Ordaz R, Sumano López H (2022). Treatment of bovine leptospirosis with enrofloxacin HCL 2H_2_O (Enro-C): a clinical trial. Animals.

[B009] Borges ALSB, Aymée L, Carvalho-Costa FA, Lilenbaum W, Di Azevedo MIN (2024). Molecular epidemiology of *Leptospira* spp. serogroup Sejroe associated with chronic bovine leptospirosis. Vet Microbiol.

[B010] Bradley EA, Lockaby G (2023). Leptospirosis and the environment: a review and future directions. Pathogens.

[B011] Carvalho HGAC, Silva DM, Rodrigues GRD, Gameiro AH, Santos RF, Raineri C, Lima AMC (2024). Estimation of economic losses due to leptospirosis in dairy cattle. Prev Vet Med.

[B012] Cidade-Brasil (2021). Município de Valença, Rio de Janeiro.

[B013] Chin VK, Basir R, Nordin SA, Abdullah M, Sekawi Z (2020). Pathology and host immune evasion during human leptospirosis: a review. Int Microbiol.

[B014] Correia L, Loureiro AP, Lilenbaum W (2017). Effects of rainfall on incidential and host-maintained leptospiral infection in cattle in a tropical region. Vet J.

[B015] Di Azevedo MIN, Lilenbaum W (2021). An overview on the molecular diagnosis of animal leptospirosis. Lett Appl Microbiol.

[B016] Dobson KJ (1974). Letter: eradication of leptospirosis in commercial pig herds. Aust Vet J.

[B017] Ellis WA (2015). Animal Leptospirosis. Curr Top Microbiol Immunol.

[B018] Furquim MEC, Santos RF, Mathias LA (2021). Antibodies against *Leptospira* spp. in bovine serum samples from several Brazilian states analyzed in the period from 2007 to 2015. Arq Bras Med Vet Zootec.

[B019] Hamond C, Martins G, Loureiro AP, Pestana C, Lawson-Ferreira R, Medeiros MA, Lilenbaum W (2014). Urinary PCR as na increasingly useful tool for an accurate diagnosis of leptospirosis in livestock. Vet Res Commun.

[B020] IBGE (2022). Brasil/Rio de Janeiro/Valença: pecuária.

[B021] INMET (2010). Normal climatológico do Brasi.

[B022] Libonati HA, Santos GB, Souza GN, Brandão FZ, Lilenbaum W (2018). Leptospirosis is strongly associated to estrus repetition on cattle. Trop Anim Health Prod.

[B023] Lilenbaum W, Souza GN (2003). Factors associated with bovine leptospirosis in Rio de Janeiro, Brazil. Res Vet Sci.

[B024] Loureiro AP, Lilenbaum W (2020). Genital bovine leptospirosis: a new look for an old disease. Theriogenology.

[B025] Martins G, Penna B, Lilenbaum W (2010). Maintenance of Leptospira infection in cattle under tropical conditions. Vet Rec.

[B026] Martins G, Lilenbaum W (2013). The panorama of animal leptospirosis in Rio de Janeiro, Brazil, regarding the seroepidemiology of the infection in the tropical regions. BMC Vet Res.

[B027] Martins G, Lilenbaum W (2017). Control of bovine leptospirosis: aspects for consideration in a tropical environment. Res Vet Sci.

[B028] Martins G, Guadelupe B, Aymée L, Balaro MFA, Pinto PH, Di Azevedo MIN, Brandão FZ, Lilenbaum W (2022). The efficacy of vaccination in the prevention of renal and genital leptospirosis in experimentally infected sheep. Trop Med Infect Dis.

[B029] Mughini-Gras L, Bonfanti L, Natale A, Comin A, Ferronato A, La Greca E, Patregnani T, Lucchese L, Marangon S (2014). Application of an integrated outbreak management plan for the control of leptospirosis in dairy cattle herds. Epidemiol Infect.

[B030] Oliveira GDM, Garcia LAN, Soares LAP, Lilenbaum W, Souza GN (2022). Leptospirosis by Sejroe strains leads to embryonic death (ED) in herds with reproductive disorders. Microbiol Spectr.

[B031] Pimenta CLRM, Costa DF, Silva MLCR, Pereira HD, Araújo JP, Malossi CD, Ullmann LS, Alves CJ, Azevedo SS (2019). Strategies of the controlo f na outbreak of leptospiral infection in dairy cattle in Northeastern Brazil. Trop Anim Health Prod.

[B032] Pinto PS, Libonati H, Penna B, Lilenbaum W (2016). A sytematic review on microscopic agglutination test seroepidemiology of bovine leptospirosis in Latin America. Trop Anim Health Prod.

[B033] Pinto PS, Barbosa C, Ferreira AMR, Lilenbaum W (2020). Uterine leptospiral infection is strongly associated to strains of serogroup Sejroe on experimentally infected hamsters. Microb Pathog.

[B034] Schoonman L, Swai ES (2010). Herd- and animal-level risk factors for bovine leptospirosis in Tanga region of Tanzania. Trop Anim Health Prod.

[B035] Sohm C, Steiner J, Jöbstl J, Wittek T, Firth C, Steinparzer R, Desvars-Larrive A (2023). A systematic review on leptospirosis in cattle: A European perspective. One Health.

[B036] Stoddard RA, Gee JE, Wilkins PP, McCaustland K, Hoffmaster AR (2009). Detection of pathogenic *Leptospira* spp. through TaqMan polymerase chain reaction targeting the LipL32 gene. Diagn Microbiol Infect Dis.

[B037] Waldmann KH (1990). Progression and controlo f leptospirosis in a sow herd. Dtsch Tierarztl Wochenschr.

[B038] WOAH (2021). WOAH manual of diagnostic tests and vaccines for terrestrial animals.

